# Untangling the Role of Capping Agents in Manipulating Electrochemical Behaviors Toward Practical Aqueous Zinc‐Ion Batteries

**DOI:** 10.1002/adma.202412790

**Published:** 2025-01-07

**Authors:** Ruwei Chen, Yunpeng Zhong, Peie Jiang, Hao Tang, Fei Guo, Yuhang Dai, Jie Chen, Jingyi Wang, Jiyang Liu, Song Wei, Wei Zhang, Wei Zong, Fangjia Zhao, Jichao Zhang, Zhengxiao Guo, Xiaohui Wang, Guanjie He

**Affiliations:** ^1^ Department of Chemistry University College London London WC1E 7JE UK; ^2^ State Key Laboratory of Pulp and Paper Engineering South China University of Technology Guangzhou 510640 China; ^3^ Department of Engineering Science University of Oxford Parks Road Oxford OX1 3PJ UK; ^4^ Department of Chemistry The University of Hong Kong Hong Kong Island Hong Kong SAR 999077 China

**Keywords:** aqueous zinc‐ion battery, capping agents, crystallographic orientation manipulation, electrode‐electrolyte interface, solvation structure

## Abstract

Long‐standing challenges including notorious side reactions at the Zn anode, low Zn anode utilization, and rapid cathode degradation at low current densities hinder the advancement of aqueous zinc‐ion batteries (AZIBs). Inspired by the critical role of capping agents in nanomaterials synthesis and bulk crystal growth, a series of capping agents are employed to demonstrate their applicability in AZIBs. Here, it is shown that the preferential adsorption of capping agents on different Zn crystal planes, coordination between capping agents and Zn^2+^ ions, and interactions with metal oxide cathodes enable preferred Zn (002) deposition, water‐deficient Zn^2+^ ion solvation structure, and a dynamic cathode‐electrolyte interface. Benefiting from the multi‐functional role of capping agents, dendrite‐free Zn plating and stripping with an improved Coulombic efficiency of 99.2% and enhanced long‐term cycling stability are realized. Remarkable capacity retention of 91% is achieved for cathodes after more than 500 cycles under a low current density of 200 mA g^−1^, marking one of the best cycling stabilities to date. This work provides a proof‐of‐concept of capping agents in manipulating electrochemical behaviors, which should inspire and pave a new avenue of research to address the challenges in practical energy storage beyond AZIBs.

## Graphical abstract

Inspired by the critical role of capping agents in nanomaterials synthesis and bulk crystal growth, the role of capping agents in manipulating the electrochemical behaviors of AZIBs is untangled. The multifunctional role of the capping agents in terms of the Zn anode, electrolyte, and cathode validates stable and practical AZIBs.

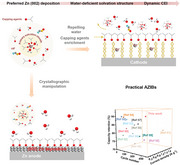

## 1 Introduction

AZIBs have attracted significant attention as a promising electrochemical energy storage solution for grid‐scale and other stationary applications in the post‐lithium era.^[^ ^1^, ^2^ ^]^ With environmental friendliness, inherent safety, high theoretical capacities (5855 mAh cm^−3^ and 820 mAh g^−1^), and low cost, they stand out among other alternatives.^[^ ^3^ ^]^ Despite these advantages, several long‐standing challenges, particularly the poor battery reversibility and stability caused by both cathodes and anodes still need to be addressed before the practical implementation of AZIBs.^[^ ^4^ ^]^

**Scheme 1:**
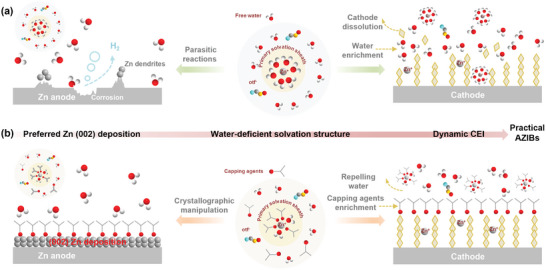
Schematic illustration of electrochemical behaviors of AZIBs in different electrolytes. a) Electrochemical behaviors of AZIBs in common aqueous electrolytes. b) The multifunctional role of capping agents and electrochemical behaviors of AZIBs in aqueous electrolytes with capping agents.

A thorough understanding of underlying challenges is essential to developing effective solutions. In typical aqueous electrolytes, Zn^2^⁺ ions are commonly solvated by six water molecules, forming [Zn(H_2_O)_6_]^2+^ complexes, along with free anions and free water molecules in the bulk electrolyte. These solvated Zn^2^⁺ ions encounter significant desolvation penalties and undergo severe parasitic reactions at the electrolyte‐electrode interface, driven by strong Coulombic interactions between the divalent Zn^2^⁺ ions and their surrounding solvation shell.^[^ ^5^, ^6^ ^]^ On the cathode side, free and co‐intercalated water molecules with strong polarity can cause irreversible lattice structure collapse, inducing severe dissolution of cathode materials and rapid capacity degradation especially at low current densities (<1C) (**Scheme** **1**a).^[^ ^7^ ^]^ On the anode side, the solvation effect drives the electron shift via Zn^2+^–OH_2_ coordination, significantly weakening the O–H bonds and accelerating the decomposition of solvated water molecules during the plating process. This, in turn, triggers the notorious surface passivation, dendrites growth, and hydrogen evolution reaction (Scheme 1a).^[^ ^8^, ^9^ ^]^ Consequently, a large excess of Zn is required to replenish the consumption caused by side reactions during cycling, highlighting the low Zn anode utilization (≈1%) when paired with typically low areal capacity cathodes (≈0.3 mAh cm^−2^) in most studies.^[^ ^10^, ^11^ ^]^ Considerable research efforts have been devoted to tackling these challenges on either the anode or cathode through electrode design, separator modification, and electrolyte optimization.^[^ ^12^ ^]^ Nevertheless, most of these studies tend to address the issues from only one electrode, and few approaches can successfully tackle these challenges simultaneously by a simple remedy.

Zn is hexagonal close‐packed metal, with (101), (002), and (100) crystal planes as its characterized surface features.^[^ ^13^ ^]^ The Zn (002) crystal plane stands out due to its relatively low surface energy and high atomic stacking density, offering superior corrosion resistance, enabling horizontal alignment of Zn deposition, and suppressing dendrites growth and side reactions.^[^ ^14^ ^]^ This makes oriented growth of Zn (002) crystal plane an effective methodology for stabilizing zinc anode.^[^ ^15^ ^]^ Unfortunately, it still encounters challenging owing to the propensity of zinc to deposit in a range of complex and random morphologies. When encountered with a similar dilemma, capping agents are often employed to control the evolution of seeds into nanocrystals with well‐defined morphologies during nanomaterials synthesis and bulk crystal growth.^[^ ^16^ ^]^ Theoretically, capping agents with multiple functional groups can selectively adsorb onto specific facets, significantly altering the surface free energy and growth dynamics. This provides a versatile strategy for maneuvering morphologies and physical/chemical properties.^[^ ^17^ ^]^ Analogously, Zn plating can be fundamentally considered as an electro‐crystallization process, during which Zn^2+^ ions in the electrolyte reduce to Zn atoms, gather into nanoscale Zn nuclei, then continuously grow into crystallized Zn metal at the microscale.^[^ ^15^ ^]^ Additionally, capping agents exhibit unique interactions with metal oxide nanomaterials and are often adopted into the synthesis process of manganese‐ and vanadium‐based cathode materials.^[^ ^18^, ^19^, ^20^, ^21^ ^]^ Consequently, capping agents hold great potentials for manipulating electrochemical behaviors in AZIBs. Although previous studies employed a few additives—ever used as capping agents in nanomaterial synthesis—to address issues associated with the Zn anode, the application of capping agents in AZIBs remains rare, let alone an in‐depth understanding of their roles in both anode, electrolyte, and cathode during the battery operation (Table S1, Supporting Information).^[^ ^22^, ^23^, ^24^, ^25^ ^]^

As a proof of concept, for the first time, the role of capping agents in AZIBs is elucidated through experimental and theoretical characterizations. Three typical and efficient capping agents for nanomaterials synthesis and bulk crystal growth—citric acid (CA), hexadecyltrimethylammonium bromide (CTAB), and polyvinyl pyrrolidone (PVP)—were employed during the Zn plating process, where parallel growth along the Zn (002) plane was promoted due to the preferential adsorption on the Zn (101) and Zn (100) planes (Scheme 1b). Featuring the polar functional groups, capping agents can replace water molecules in the solvation structure of Zn^2+^ ions, forming water‐deficient solvation structures and alleviating water‐induced side reactions (Scheme 1b). Consequently, dendrite‐free Zn plating/stripping with an improved Coulombic efficiency of 99.2% and an enhanced long‐term cycling stability (>700 h) at a large areal capacity of 10 mAh cm^−2^ was realized. Furthermore, capping agents also act as ligands and form complexes with metal oxide cathodes, which repel free water molecules and prevent the intercalation of hydrated Zn^2+^ ions, effectively suppressing the cathode dissolution and guaranteeing reversible ion (de)intercalation (Scheme 1b). As a result, a remarkable capacity retention of 91% was achieved for cathodes after more than 500 cycles under a low current density of 200 mA g^−1^, representing one of the best cycling stabilities to date. Benefiting from the multifunctional role of capping agents in terms of anode, electrolyte, and cathode, the practical high‐areal‐capacity (2.39 mAh cm^−2^) full cell with a high Zn utilization (N/P = 2.5) operated stably for over 230 cycles (Scheme 1b). This work provides a holistic perspective on the roles of capping agents throughout the entire battery system, which will advance the understanding of capping agents and arouse new inspiration for broader applications in practical energy storage beyond AZIBs.

## 2 Results and Discussion

**Figure 1:**
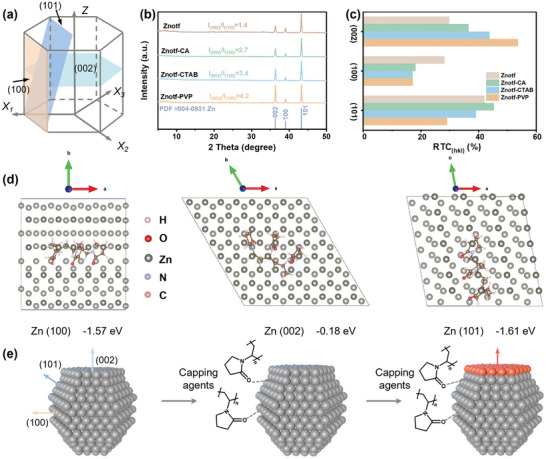
The role of capping agents on the Zn anode. a) Illustration of the hexagonal close‐packed structure of Zn. b) GIXRD patterns of Zn anodes cycled in various electrolytes. c) RTC values of cycled Zn in various electrolytes. d) Theoretical models and corresponding adsorption energy of the selected capping agent on Zn (100), Zn (002), and Zn (101) crystal planes. e) Schematic illustration of Zn deposition evolution with capping agents.

Zn is hexagonal close‐packed metal with (101), (002), and (100) crystal planes dominated crystallographic features (**Figure** **1**a), in which the (002) crystal plane shows relatively high resistance to corrosion and dendrites growth.^[^ ^26^ ^]^ However, Zn deposition in bare Znotf electrolyte tends to form complex and random morphologies, with an I_002_/I_100_ ratio of 1.4 (Figure 1b; Figure S1a,b, Supporting Information). In contrast, continuously increased I_002_/I_100_ ratios are observed in modified electrolytes containing capping agents, such as CA, CTAB, and PVP (Figure S2, Supporting Information), demonstrating the versatile role of capping agents in promoting the preferential growth of the Zn deposition toward the (002) crystal plane (Figure 1b; Figure S1c–h, Supporting Information). To provide additional insight into the evolution of Zn texture, relative texture coefficients (RTC) were calculated, where a larger RTC value indicates a more pronounced crystal texture.^[^ ^27^ ^]^ In comparison to Znotf electrolyte, the (002) RTC values increase from 29.8 to 36.5, 43.8, and 53.7 for Znotf‐CA, Znotf‐CTAB, and Znotf‐PVP electrolytes, respectively (Figure 1c). Among them, PVP shows a significant increase in the (002) RTC value, revealing its effectiveness in manipulating the Zn (002) crystal texture (Table S2, Supporting Information). Thus, PVP is selected as the representative capping agent for further discussion.

To understand the preferential growth mechanism induced by capping agents, the interactions between the selected capping agent and various Zn crystal planes were performed using density functional theory (DFT) calculations. The binding affinity of capping agents toward different crystal planes is influenced by the specific surface energy of each lattice plane.^[^ ^16^, ^28^ ^]^ The Zn (002) crystal plane, with its higher atomic stacking density, has the lowest surface energy compared with the Zn (101) and Zn (100) planes.^[^ ^29^, ^30^ ^]^ As shown in Figure 1d, the adsorption energy between the capping agent and the Zn (002) plane (−0.18 eV) is greater than that for the Zn (101) plane (−1.61 eV) and the Zn (100) plane (−1.57 eV), indicating that the capping agent preferentially adsorbs on the Zn (101) and Zn (100) crystal planes (Figure S3, Supporting Information).^[^ ^31^ ^]^ FTIR spectra also give experimental evidence for the adsorption of the capping agent on the Zn surface (Figure S4, Supporting Information). Owing to the selectivity of the capping agent toward the Zn (101) and Zn (100) crystal planes, Zn deposition on these planes is retarded, thereby promoting the preferred deposition on the Zn (002) plane (Figure 1e).

**Figure 2:**
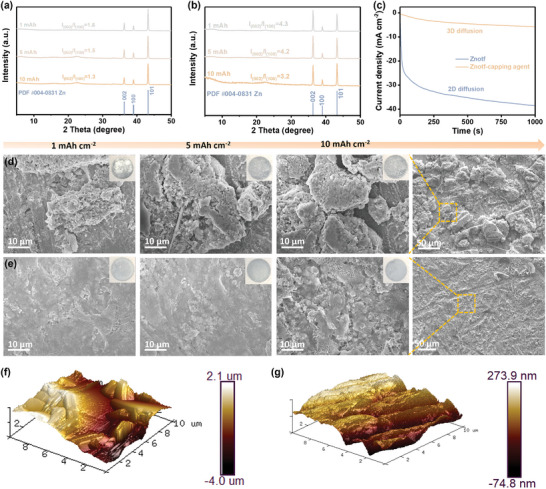
Evolution of Zn deposition texture in different electrolytes. XRD patterns of Zn deposition at an areal current density of 1 mA cm^−2^ for varying areal capacities in (a) the Znotf electrolyte and (b) the Znotf‐capping agent electrolyte. c) Chronoamperometry curves in different electrolytes. SEM images of Zn deposition at an areal current density of 1 mA cm^−2^ for varying areal capacities in (d) the Znotf electrolyte and (e) the Znotf‐capping agent electrolyte. Insets display corresponding optical images. f) AFM image of the Zn surface after cycling in the Znotf electrolyte. g) AFM image of the Zn surface after cycling in the Znotf‐capping agent electrolyte.

To further evaluate the capability of the capping agent in manipulating crystallographic orientation, grazing incident X‐ray diffraction (GIXRD) was used to verify the texture evolution of Zn deposition with prolonged plating capacities. In the Znotf electrolyte, the diffraction peak intensity ratios of I_002_/I_100_ are very low at various plating capacities (**Figure** **2**a). Scanning electron microscope (SEM) images show that Zn deposition tends to form large dendrites with irregular and nonplanar morphologies when the deposition capacity rises from 1 to 10 mAh cm^−2^ (Figure 2d; Figure S5a–c, Supporting Information). In comparison, the I_002_/I_100_ ratio obviously increases in the Znotf‐capping agent electrolyte (Figure 2b). Zn deposition in this electrolyte stacks horizontally, forming an evenly distributed and compact structure (Figure 2e; Figure S5d–f, Supporting Information). A remarkable I_002_/I_100_ ratio of 3.2 and a uniform, flat surface are maintained even under a large plating capacity of 10 mAh cm^−2^, demonstrating the effectiveness of the capping agent in promoting Zn (002) crystal plane growth and uniform Zn deposition. As shown in chronoamperometry curves (Figure 2c), Zn deposition in the Znotf electrolyte shows a consistent 2D diffusion process characterized by a rapid current increase, demonstrating the uncontrolled growth of a porous Zn deposition layer and a rapid increase of the specific surface area.^[^ ^32^ ^]^ Conversely, the Zn deposition in the Znotf‐capping agent electrolyte quickly transitions to a steady 3D diffusion process with a low and stable current, indicating a uniform Zn deposition layer.^[^ ^33^ ^]^ This finding aligns with the evolution of morphologies observed in the SEM images. Additionally, atomic force microscopy (AFM) images also reveal greatly reduced surface roughness of the cycled Zn electrode in the Znotf‐capping agent electrolyte (Figure 2f,g; Figure S6, Supporting Information). These observations strongly support the effective capability of the capping agent in crystallographic orientation manipulation.

**Figure 3:**
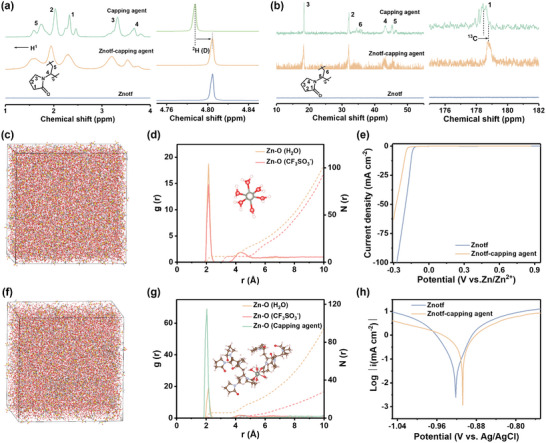
Evolution of the electrolyte environment. a) ^1^H NMR spectra of different electrolytes. b) ^13^C NMR spectra of different electrolytes. c) 3D snapshot of the Znotf electrolyte obtained from MD simulations. d) Corresponding RDF g(r) and coordination number N(r). e) LSV curves in different electrolytes. f) 3D snapshot of the Znotf‐capping agent electrolyte obtained from MD simulations. g) Corresponding RDF g(r) and coordination number N(r). h) Tafel plots for different electrolytes.

The electrolyte environment is another crucial factor affecting the performance of AZIBs. In the ^1^H nuclear magnetic resonance (NMR) spectra (**Figure** **3**a), the ^2^H peak of D_2_O in the capping agent solution is located at 4.79 ppm, where this peak shows a downfield chemical shift upon the addition of Znotf salt, indicating the water‐coordinated solvation structure in both electrolytes.^[^ ^34^ ^]^ The coordination between Zn^2+^ and D_2_O impels the electron shift from hydrogen atoms to oxygen atoms, resulting in the reduced electron density around hydrogen atoms. Meanwhile, all ^1^H peaks corresponding to capping agent experience upfield chemical shift in the Znotf‐capping agent electrolyte, revealing an enhanced shielding effect due to the interaction between Znotf and the capping agent.^[^ ^35^ ^]^ From the ^13^C NMR spectra, the peak at 178.46 ppm indexes to C‐1 in the pyrrolidone ring of the capping agent, which shifts toward lower magnetic field upon the addition of Znotf (Figure 3b).^[^ ^36^ ^]^ This shift suggests reduced electron density and enhanced de‐shielding effect on the C‐1, attributed to the interaction of oxygen (─C≐O) in the capping agent with Zn^2+^ ions.^[^ ^37^ ^]^ As shown in FTIR spectra (Figure S7, Supporting Information), the Zn‐O stretching at 636 cm^−1^ and the C≐O stretching at 1646 cm^−1^ shift to lower wavenumbers, also indicating the presence of the interaction between the capping agent and Zn^2+^ ions. These results indicate that the capping agent can participate in the solvation structure of Zn^2+^ ions.

Molecular dynamics (MD) simulations were conducted to explore the solvation structure of Zn^2+^ ions. The Zn‐O radial distribution functions (RDFs) reveal that an average coordination number of water molecules around Zn^2+^ ions is 5.9 in the Znotf electrolyte, showing a predominant Zn(H_2_O)_6_^2+^ solvation structure (Figure 3c,d).^[^ ^38^ ^]^ In comparison, a new peak corresponding to Zn‐O (capping agent) appears in the Znotf‐capping agent electrolyte. The average coordination number between water molecules and Zn^2+^ ions decreases to 2.4, indicating that capping agent molecules can replace water molecules and participate in Zn^2+^ ion solvation structures (Figure 3f,g). These results are consistent with the above‐mentioned NMR analysis. This water‐deficient Zn^2+^ ions solvation structure is beneficial for inhibiting water‐induced side reactions, as evidenced by the lower hydrogen evolution overpotential, lower corrosion current, higher corrosion potential, and in situ observed dendrite‐free Zn plating behavior in the Znotf‐capping agent electrolyte (Figure 3e,h; Figure S8, Supporting Information).

**Figure 4:**
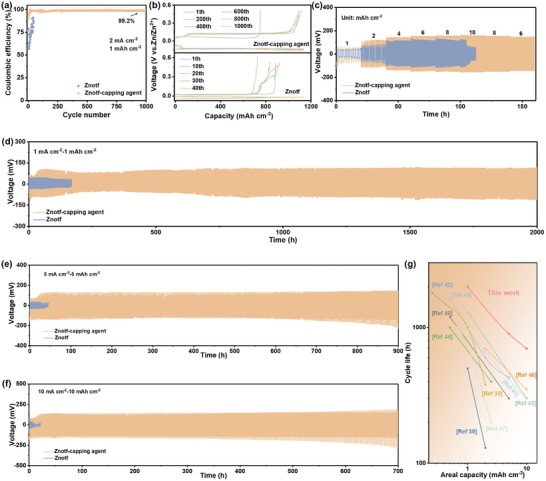
Zn plating/stripping behaviors in different electrolytes. a) Comparison of Coulombic efficiency. b) Corresponding voltage profiles. c) Rate performance at various current densities. Long‐term cyclic performance of Zn||Zn symmetric cells at d) 1 mA cm^−2^ and 1 mAh cm^−2^, e) 5 mA cm^−2^ and 5 mAh cm^−2^, and f) 10 mA cm^−2^ and 10 mAh cm^−2^. g) Comparison of the cyclic life of this work with recent studies.

Benefiting from the synergistic effect of crystallographic orientation manipulation and water‐deficient Zn^2+^ ion solvation structure, highly reversible and stable Zn plating/stripping behaviors are predictable for AZIBs in the Znotf‐capping agent electrolyte. After screening the effect of capping agent concentration on Zn plating/stripping behaviors, the reversibility of Zn plating and stripping is assessed in Zn||Cu half cells under a constant current density of 2 mA cm^−2^ and a capacity of 1 mAh cm^−2^ (Figure S9, Supporting Information). An inferior average Coulombic efficiency (CE) of 76.2% with fluctuating voltage profiles and a limited lifespan below 100 cycles is observed in the Znotf electrolyte, indicating severe side reactions and poor reversibility (**Figure** **4**a,b). The cell with the Znotf‐capping agent electrolyte achieves a significantly improved average CE of 99.2% and steady voltage profiles over 1000 cycles are obtained, indicating excellent reversibility and substantially suppressed side reactions. The capping agent is also effective in the ZnSO_4_ electrolyte, further proving its versatile role in manipulating Zn plating/stripping behaviors (Figure S10, Supporting Information). In addition, the rate performance of Zn||Zn symmetric cells is also studied at various current densities and areal capacities from 1 mA cm^−2^–1 mAh cm^−2^ to 10 mA cm^−2^–10 mAh cm^−2^ (Figure 4c). A sudden short circuit occurs in the cell with the Znotf electrolyte at 10 mA cm^−2^−10 mAh cm^−2^. On the contrary, the Zn||Zn symmetric cell in the Znotf‐capping agent electrolyte delivers stable voltage profiles across all current densities and areal capacities, which highlights the stable and reversible Zn plating/stripping behaviors under various conditions.

The long‐term cyclic stability is further evaluated in Zn||Zn symmetric cells under various current densities and areal capacities. With the Znotf‐capping agent electrolyte, Zn||Zn symmetric cells demonstrate stable cycling for over 2000 and 900 h at 1 mA cm^−2^–1 mAh cm^−2^ and 5 mA cm^−2^–5 mAh cm^−2^, respectively (Figure 4d,e; Figure S11, Supporting Information). Remarkably, even at a high current density of 10 mA cm^−2^ and a high capacity of 10 mAh cm^−2^, the cell still maintains an impressive cycle life of over 700 h (Figure 4f). Impressively, the Zn||Zn symmetric cell using ultrathin Zn electrodes (10 µm) can still deliver the stability for more than 160 h at a high depth of discharge (DOD) of 70%, which is comparable to other recently reported studies (Table S3 and Figure S12, Supporting Information). The cycling performance achieved in this work surpasses most recently reported state‐of‐the‐art studies, which strongly supports the effective capability of the capping agent at the Zn anode side (Figure 4g).^[^ ^33^, ^39^, ^40^, ^41^, ^42^, ^43^, ^44^, ^45^, ^46^, ^47^ ^]^

**Figure 5:**
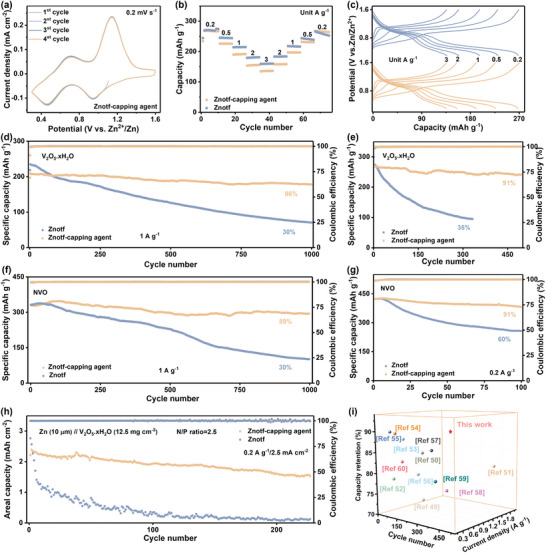
Electrochemical performances of full cells and universal applications of the capping agent. a) CV curves of Zn||V_2_O_5_.xH_2_O batteries in the Znotf‐capping agent electrolyte. b) Rate performance of Zn||V_2_O_5_.xH_2_O batteries in different electrolytes. c) Corresponding voltage profiles. d,e) Cyclic performances of Zn||V_2_O_5_.xH_2_O batteries in different electrolytes at 1 and 0.2 A g^−1^, respectively. f,g) Cyclic performances of Zn||NVO batteries in different electrolytes at 1 and 0.2 A g^−1^, respectively. h) Cyclic performances of practical Zn||V_2_O_5_.xH_2_O batteries with a high mass loading and a low N/P ratio. i) Comparison of cyclic stability at low current densities with other recently reported full batteries.

Full cells using Zn metal anodes and different cathodes were assembled to evaluate the capping agent's universality for practical applications. As shown in **Figures** **5**a and S13 (Supporting Information), cyclic voltammetry (CV) curves of Zn||V_2_O_5_.xH_2_O batteries in both electrolytes exhibit two pairs of redox peaks, which are attributed to the Zn^2+^/H^+^ co‐insertion mechanism and consistent with previous works.^[^ ^48^ ^]^ Notably, the Zn||V_2_O_5_.xH_2_O battery in the Znotf‐capping agent electrolyte displays more steady CV profiles compared with the Znotf electrolyte, demonstrating improved reversibility and stability due to the capping agent. Figure 5b and Figure S14 (Supporting Information) compare the rate performance of Zn||V_2_O_5_.xH_2_O batteries in different electrolytes, where a better rate performance is achieved in the Znotf electrolyte as current densities and scan rates increase stepwise (Figure 5c). Although the initial rate performance of the cell in the Znotf‐capping agent electrolyte is lower than the one in the Znotf electrolyte, the final capacity of the cell in the Znotf‐capping agent electrolyte is higher when the current density returns to 0.2 A g^−1^, which is due to the rapid capacity decay of the Zn||V_2_O_5_.xH_2_O battery in the Znotf electrolyte (Figure 5b).

The long‐term cyclic performance was further studied. As shown in Figure 5d, the Zn||V_2_O_5_.xH_2_O battery in the Znotf‐capping agent electrolyte maintains a high‐capacity retention of 89% after 1000 cycles at 1 A g^−1^. In contrast, rapid capacity fading with low‐capacity retention of 30% is observed for the Zn||V_2_O_5_.xH_2_O battery in the Znotf electrolyte. These results demonstrate the critical role of the capping agent in improving cathode stability. In particular, an impressive capacity retention of 91% is achieved for the Zn||V_2_O_5_.xH_2_O battery in the Znotf‐capping agent electrolyte after 500 cycles even at a low current density of 0.2 A g^−1^, marking one of the best cyclic performances at low current densities to date (Figure 5e,i).^[^ ^49^, ^50^, ^51^, ^52^, ^53^, ^54^, ^55^, ^56^, ^57^, ^58^, ^59^, ^60^ ^]^ Moreover, we applied the Znotf‐capping agent electrolyte to other vanadium oxide and manganese oxide cathodes. The significantly improved cyclic performance can also be achieved at both high and low current densities, highlighting the generalizability of the capping agent (Figure 5f,g; Figure S15, Supporting Information). To assess the feasibility of the capping agent under practical conditions, full cells with a low N/P ratio of 2.5 were assembled by using ultrathin Zn anodes (10 µm) and high mass loading cathodes (12.5 mg cm^−2^). This configuration presents a significant challenge because both stable Zn anode with high utilization and reversible cathode with high mass loading are required. The assembled full cell with capping agent delivers a high areal capacity of 2.39 mAh cm^−2^ and significantly enhanced cyclic stability after 230 cycles at 0.2 A g^−1^. In contrast, fast capacity fading with noticeable CE fluctuation is observed in the cell with the Znotf electrolyte, highlighting the excellent effectiveness of the capping agent for both anode and cathode (Figure 5h).

**Figure 6:**
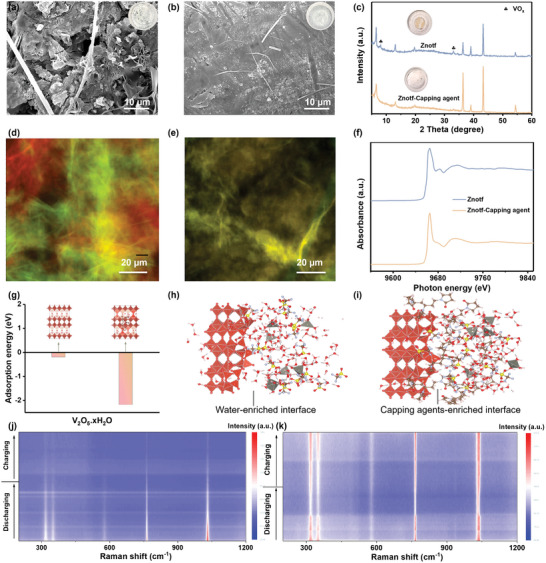
Exploration of the mechanism for significantly improved stability. a,b) SEM images of Zn anodes disassembled from full cells after long‐term cycling in Znotf and Znotf‐capping agent electrolytes, respectively. Insets display the corresponding optical images. c) XRD patterns of Zn anodes disassembled from full cells after long‐term cycling in different electrolytes. Insets show the corresponding optical images of cycled separators. d,e) Synchrotron XRF mapping of long‐term cycled V_2_O_5_.xH_2_O cathodes in Znotf and Znotf‐capping agent electrolytes, respectively. Green represents Zn element, red represents V element, and yellow represents mixed Zn‐V elements. f) Corresponding Zn K‐edge XANES spectra extracted from (d,e). g) Adsorption energy of electrolyte components on the cathode. h,i) Snapshots of the electrolyte‐cathode interface at the end stage in Znotf and Znotf‐capping agent electrolytes, respectively. j,k) In situ Raman spectra of V_2_O_5_.xH_2_O cathodes in Znotf and Znotf‐capping agent electrolytes, respectively.

The underlying mechanism for the superior cyclic stability of full cells was further investigated. On the anode, a rough and corroded Zn surface with obvious dendrites is observed in the Znotf electrolyte after long‐term cycling, which disrupts the reversibility of overall electrochemical reaction and accelerates cell degradation (**Figure** **6**a).^[^ ^61^ ^]^ By taking advantage of the synergistic effect of crystallographic orientation manipulation and water‐deficient Zn^2+^ ion solvation structure, the Zn surface remains uniform and planar after long‐term cycling in the Znotf‐capping agent electrolyte. This suggests greatly inhibited side reactions at the anode/electrolyte interface, benefiting overall battery performances (Figure 6b).^[^ ^62^ ^]^ On the cathode side, the cycled cathode in the Znotf‐capping agent electrolyte retains a similar structure and morphology to the pristine cathode (Figure S16a–c,g–i, Supporting Information). In sharp contrast, the cycled cathode in the Znotf electrolyte shows significant morphological changes due to severe cathode dissolution (Figure S16d–f, Supporting Information). This is further corroborated by the detection of a VO_x_ signal on the surface of the Zn anode and the observed color change of the separator in the Znotf electrolyte (Figure 6c).

Synchrotron X‐ray fluorescence (XRF) mapping was employed to gain deeper insights into the evolution of the cathode in different electrolytes after long‐term cycling. Fluorescence maps were taken at the end of the charging process. From the Zn and V map of the cathode in the Znotf electrolyte, distinct boundaries between Zn and V elements are observed (Figure 6d). The extracted X‐ray Absorption Near Edge Structure (XANES) spectroscopic data from the red region indicates the presence of Zn_x_(otf)_y_(OH)_2x−y_·nH_2_O species, which may be formed in situ on the cathode as a result of irreversible (de)intercalation of H^+^ (Figure 6f).^[^ ^63^, ^64^ ^]^ These results show obvious Zn_x_(otf)_y_(OH)_2x−y_·nH_2_O species in the cathode composition, which is consistent with the SEM results and suggests the severe cathode dissolution and irreversible ion (de)intercalation (Figure S16d–f, Supporting Information). Impressively, the XRF map of the cathode in the Znotf‐capping agent electrolyte shows an overlapped distribution of Zn and V elements (Figure 6e). The corresponding XANES spectrum displays a distinct signal of Zn‐V composite rather than Zn_x_(otf)_y_(OH)_2x−y_·nH_2_O, confirming the stable cathode structure and reversible ion (de)intercalation aided by capping agent (Figure 6f).^[^ ^65^ ^]^

DFT and MD simulations were performed to provide theoretical insights into the effect of the capping agent on the cathode. The absorption energy of the capping agent on the cathode material was calculated to be −2.18 eV, higher than that of water molecules (−0.202 eV) (Figure 6g). This indicates that capping agent molecules can absorb on the cathode surface, forming a capping agent‐enriched dynamic CEI (Figure 6i). Compared with the water‐enriched interface in Znotf electrolyte, the dynamic CEI combined with water‐deficient Zn^2+^ ion solvation structures can repel free water and prevent the intercalation of hydrated Zn^2+^ ions, thus effectively suppressing the cathode dissolution and enabling reversible ion (de)intercalation (Figure 6h; Figure S17, Supporting Information). The stability and reversibility of this process were verified by in situ Raman spectra obtained at low current densities. Raman peaks located at 310–360 and 574 cm^−1^ correspond to the bending mode of the V–O–V bonds, while the peaks appeared at 760 and 1031 cm^−1^ index to the stretching mode of the V≐O bond.^[^ ^6^, ^66^ ^]^ In the Znotf‐capping agent electrolyte, these peaks gradually weaken during the discharge process and subsequently recover during the charge process, proving reversible phase changes and ion (de)intercalation (Figure 6k). However, the gradually weakened peaks cannot recover and even disappear of the cathode in the Znotf electrolyte, suggesting irreversible transitions due to severe cathode dissolution (Figure 6j). Hence, the multifunctional role of the capping agent in terms of the Zn anode, electrolyte, and cathode validates stable and practical AZIBs.

## 3 Conclusion

In summary, this study provides a proof‐of‐concept of capping agents in manipulating the electrochemical behaviors of AZIBs. We demonstrate that the preferential adsorption of capping agents on different Zn crystal planes, coordination between capping agents and Zn^2+^ ions, and interactions with metal oxide cathodes enable preferred Zn (002) deposition, water‐deficient Zn^2+^ ion solvation structure, and a dynamic CEI, thereby simultaneously tackling long‐standing challenges faced by both the anode and the cathode. Benefiting from the multifunctional role of capping agents, Zn plating/stripping with an improved CE of 99.2% and long‐term cycling stability were realized. A high‐capacity retention of 91% was achieved for cathodes after more than 500 cycles at a current density of 200 mA g^−1^. Our findings should inspire a new avenue of research activities to address challenges in practical energy storage not limited to AZIBs.

## Conflict of Interest

The authors declare no conflict of interest.

## Supporting information



Supporting Information

## Data Availability

The data that support the findings of this study are available from the corresponding author upon reasonable request.
