# Supplementary material for: Untangling the Role of Capping Agents in Manipulating Electrochemical Behaviors Toward Practical Aqueous Zinc‐Ion Batteries
Source: Adv Mater. 2025 Jan 7;37(46):2412790. doi: 10.1002/adma.202412790 (PMC12631510; doi:10.1002/adma.202412790)
Supplement: Supplementary file 1 — Supporting Information [file ADMA-37-2412790-s001.docx]

**Supporting Information**

**Untangling the Role of Capping Agents in Manipulating Electrochemical Behaviors towards Practical Aqueous Zinc-Ion Batteries**

Ruwei Chen^1#^, Yunpeng Zhong^1#^, Peie Jiang^1^, Hao Tang^2^, Fei Guo^1^, Yuhang Dai^4^, Jie Chen^1^, Jingyi Wang^1^, Jiyang Liu^1^, Song Wei^1^, Wei Zhang^1^, Wei Zong^4^, Fangjia Zhao^1^, Jichao Zhang^1^, Zhengxiao Guo^3^, Xiaohui Wang^2^*, and Guanjie He^1^*

*^1^ Department of Chemistry, University College London, London WC1E 7JE, UK*

*^2^ State Key Laboratory of Pulp and Paper Engineering, South China University of Technology, Guangzhou 510641, China*

*^3^ Department of Chemistry, The University of Hong Kong, Hong Kong Island, Hong Kong SAR, China*

*^4^ Department of Engineering Science, University of Oxford, Parks Road, Oxford OX1 3PJ, UK*

* Corresponding author email: [fewangxh@scut.edu.cn](mailto:fewangxh@scut.edu.cn); [g.he@ucl.ac.uk](mailto:g.he@ucl.ac.uk)

^#^ *These authors contributed equally to this work.*

**Experimental Section**

***Electrolyte preparation***: The Znotf electrolyte was prepared by dissolving 0.2 mol Zn(CF_3_SO_3_)_2_ (Sigma-Aldrich) into 100 mL deionized water. The Znotf-capping agents electrolyte was then prepared by dissolving 3wt% of capping agents like citric acid (Sigma-Aldrich), hexadecyltrimethylammonium bromide (Sigma-Aldrich), and polyvinyl pyrrolidone (VWR Chemicals BDH®, M_w_=700,000) into as-prepared Zn(CF_3_SO_3_)_2_ solution. All chemicals were used as received. Additionally, different amounts of polyvinyl pyrrolidone (0.5wt%, 1wt%, 2wt%, and 3wt%) were dissolved into Zn(CF_3_SO_3_)_2_ solution to investigate the effect of concentration on Zn plating/stripping behaviors.

***Synthesis of V_2_O_5_.xH_2_O***: V_2_O_5_.xH_2_O was synthesized according previous work.^[1]^ In a typical synthesis process, 0.25 g of V_2_O_5_ powder was added to a mixture of deionized water (3.85 g) and hydrogen peroxide solution (30 wt%, 1.15 g). After stirring for 15 minutes, 20 g of deionized water was added, and the solution was thoroughly mixed again. The homogeneous dark red solution was then sonicated for 2 h, followed by freeze drying to obtain V_2_O_5_.xH_2_O.

***Synthesis of NH_4_V_4_O_10_***: NH_4_V_4_O_10_ was prepared by a single-step hydrothermal reaction according to our previous work.^[2]^ Specifically, 5 mmol of ammonium metavanadate was dissolved into 30 ml of deionized water with 10 min stirring. After that, 6 mmol of oxalic acid and 1 mmol of ammonium fluoride were added. Then, the mixed solution was transferred into 50 ml Teflon-lined autoclaves and heat up to 180 ℃ for 6 hours. The as-obtained bronze-color precipitates were washed by deionized water for several times. Finally, washed precipitates were freeze-dried for 2 days to obtain NH_4_V_4_O_10_, which is denoted as NVO.

***Electrode preparation***: The cathodes were fabricated by mixing polyvinylidene fluoride (Sigma-Aldrich), Super-P (Sigma-Aldrich) and as-prepared cathode materials with a ratio of 1:2:7 using N-methyl-2-pyrrolidone as the solvent. The mixture slurry was printed on a hydrophilic carbon paper and then transferred to a vacuum oven to dry under 60 ℃ for 10 hours. The mass loading of as-fabricated cathodes was about 1~2 mg·cm^−2^. The high mass loading cathodes were prepared by mixing polytetrafluoroethylene (PTFE, 60 wt% in H_2_O, Sigma-Aldrich), Super-P, and V_2_O_5_.xH_2_O with a ratio of 1:1:8 using isopropanol (Sigma-Aldrich) as solvent. Then, the slurry was pressed by roller to form free-standing film. After drying at 60 °C, the film was cut into small pieces and pressed onto titanium mesh.

***Materials characterizations***: SEM was conducted on JEOL-JSM-6700F. The XRD patterns were performed on a PANalytical Empyrean device with Cu Kα radiation. Raman spectra were collected on Bruker Senterra II Raman Specrometer with a 532 nm laser. A Bruker dimension Icon with Scanasyst device was employed to conduct AFM experiments. The in situ optical observation was conducted on VisiScope® BL254 T1 (VWR) instrument. The XRD patterns were performed on a PANalytical Empyrean device with Cu Kα radiation. The relative texture coefficients (RTC) of the three typical Zn crystal planes are calculated by the following equation:

$${RTC}_{(hkl)}=\frac{I_{(hkl)}/I_{0(hkl)}}{\sum(I_{(hkl)}/I_{0(hkl)})}\times100$$

where I_(hkl)_ and I_0(hkl)_ are the intensity acquired from the XRD pattern of the deposited Zn electrode and standard sample.

The X-ray fluorescence mapping experiment was conducted at the resolution X-ray spectroscopy beamline (I14) at Diamond Light source. The beamline images the source in the vertical direction but images a secondary source in the horizontal. The layout consists of two mirrors at the start of the beamline which are used to shape the beam and for harmonic rejection. These mirrors focus the beam in the horizontal onto a secondary source at 50m from the source and a horizontally deflecting monochromator is used for maximum stability. I14 is a long beamline, extending over 185m with the nano-focussing KB endstation housed in an external building. The incident X-ray energy was 12 keV, focused onto the sample by a set of Kirkpatrick–Baez mirrors with a spot size of ~0.15 µm. XRF experiment was conducted by raster scanning the sample against the incident X-ray beam, and the full XRF spectrum from the sample at each scanned point (pixel) was collected by a silicon drift detector. The elemental distribution evolution can be obtained using XRF microscopy by fitting the fluorescence spectra using the MANTiS and DAWN software.

***Electrochemical measurements***: The electrochemical performances of Zn//Zn symmetric cells, Zn//Cu asymmetric cells, and full cells were tested using CR 2025 coin cells. The evaluation of cyclic voltammetry, chronoamperometry measurement, and linear sweep voltammetry tests were achieved by a VMP3 Biologic potentiostat. The evaluation of galvanostatic charge-discharge was tested by a Neware battery testing system. Glass-fiber (GF/D, Whatman) was chosen as the separator. All Zn foil (99.9%) anodes were utilized without treatment. Linear polarization curves were tested using Zn plates as the working electrode, Pt as the counter electrode, and Ag/AgCl as the reference electrode at a scan rate of 5 mV s^-1^. To assemble Zn//Zn symmetric cells with high DOD and full cells with low N/P ratio, ultrathin Zn foil (10 µm) was employed.

***Computational Details:*** MD simulations were carried out using the LAMMPS package^[3]^. The OPLS-AA force field^[4]^ with fitted parameters for otf^-^ anions and PVP molecules (from LigParGen) were used in this work. Water molecules were simulated with SPC/E model^[5]^. Parameter fitting was performed using the density functional theory (DFT) package Gaussian16^[6]^ under B3LYP exchange-correlation functional with Grimme’s DFT-D3(BJ) empirical dispersion correction and the 6-31+G** basis set was adopted for self-consistent field (SCF) calculations. The RESP2(0.5) [Non-bonded force field model with advanced restrained electrostatic potential charges (RESP2)] charge combining gas- and liquid-phase (implicit solvent, SMD model) charges were adopted for all molecules in this work calculated using Gaussian16 and Multiwfn package^[7]^. The electrolyte system contains 594 Zn^2+^ cations, 1188 otf^-^ anions and 13275 H_2_O for the baseline system, and 567 Zn^2+^ cations, 1134 otf^-^ anions, 12676 H_2_O and 24 PVP for experimental samples. The electrolyte system was initialized using the Packmol package and the Moltemplate package. The MD simulation was firstly performed in the NVT ensemble at temperature (298 K) by using 2 fs time step for 1 ns. Then equilibrium simulation is performed in the NPT ensemble at constant pressure (1 bar) and temperature (298 K) in a cubic box with periodic boundary conditions in all xyz Cartesian directions by using 2 fs time step for 2 ns. Finally, the production simulation is performed in the NVT ensemble at temperature (298 K) by using 2 fs time step for 10 ns. Electrostatic interactions are treated using the Particel-Particle-Particle-Mesh (PPPM) method. The coordination number of molecules of type i in the first solvation shell surrounding a single molecule of type j is calculated as:

$N_{i}=4\pi n_{j}\int_{0}^{R_{M}} g_{ij}(r)r^{2}dr$ (1)

in which 𝑅𝑀 is the distance of the first minimum following the first peak in the radial distribution function (RDF), and 𝑔𝑖𝑗(𝑟)is a standard approach for bulk liquid. All the visualizations of MD simulations are implemented by VMD software.

All first principle calculation (Ab initio) calculations were accomplished using Vienna Ab initio simulation package (VASP) ^[8]^. The generalized gradient approximation (GGA) functionals were considered by PerdewBurke-Ernzerh as well as projector augmented wave method (PAW-PBE) ^[9]^ to describe exchange-correlation effects between electrons. A 20.0-Å vacuum layer was introduced on the Z-axis direction to limit interactions between periodic images. The supercells with four-layer Zn slabs were chosen to represent the adsorbed surface for molecules, and the bottom two layers were fixed to maintain the bulk property. The energy cutoff for the plane-wave basis set was 520 eV. In the optimization process, the convergence criterion for the electronic self-consistent field (SCF) and loop was set to 1×10^-6^ eV/atom, in the single energy process the convergence criterion for the electronic self-consistent field (SCF) and loop was set to 1×10^-8^ eV/atom. All the structures are relaxed until the residual forces on the atoms have declined to less than 0.02 eV Å-1. For all calculations, Van der Waals interaction was taken into account at DFT-D4^[10]^. The adsorption energy is calculated via:

E_ads_ = E_Zn+mol_-E_Zn_-E_mol_ (2)

where the E_Zn+mol_, the E_Zn_, and the E_mol_ represent the total energy of adsorbates on the Zn surface, the energy of Zn surface, and the energy of the isolated adsorbates, respectively^[11]^.

The ground-state structural and electronic properties of V_2_O_5_ are obtained using DFT and (AIMD). using a 400-eV plane-wave kinetic energy cutoff. Arotationally invariant DFT+U approach is employed to describe the on-site Coulomb interaction of the spin-up and spin-down electrons, with U = 3.1 eV^[12]^. The number ratios of Zn, PVP, otf- and H_2_O are kept with the actual situation and reduced in equal proportions. The temperature of NVT ensemble was controlled using the Nose’-Hoover themostat. The

systems were first equilibrated at a higher temperature (1000 K) to generate configurations with fully mixed solvents and solutes. Then the equilibrated solution structures are used as the initial configurations for MD simulations at room temperature (298 K).


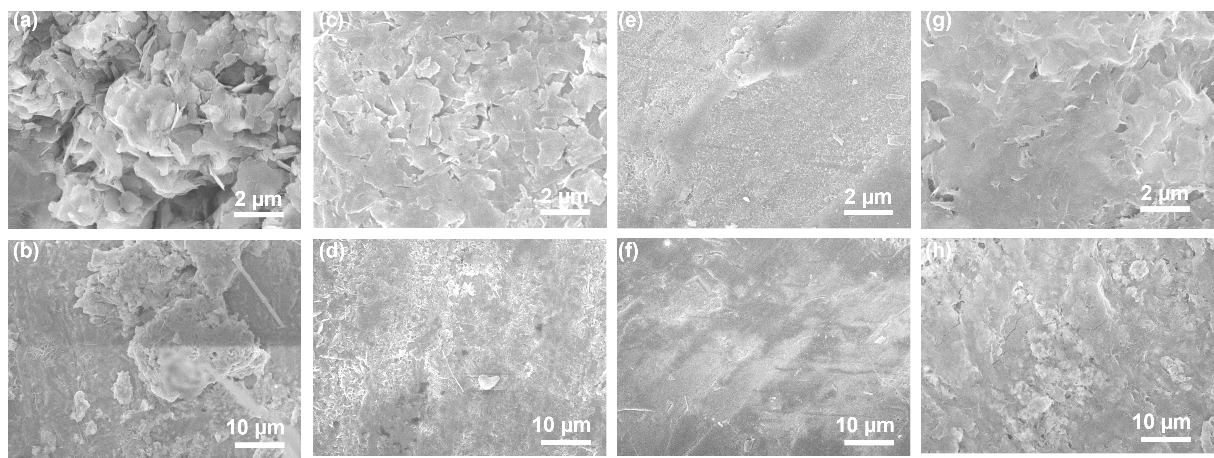


**Figure S1.** (a-b) SEM images of Zn deposition in Znotf electrolyte at different magnifications. (c-d) SEM images of Zn deposition in Znotf-CA electrolyte at different magnifications. (e-f) SEM images of Zn deposition in Znotf-CTAB electrolyte at different magnifications. (g-h) SEM images of Zn deposition in Znotf-PVP electrolyte at different magnifications.


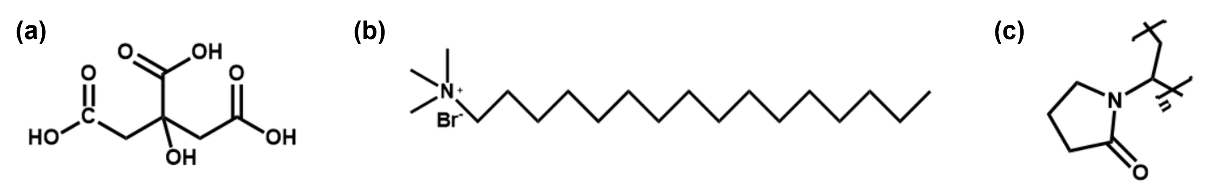


**Figure S2.** (a) Molecular structure of CA. (b) Molecular structure of CTAB. (c) Molecular structure of PVP.


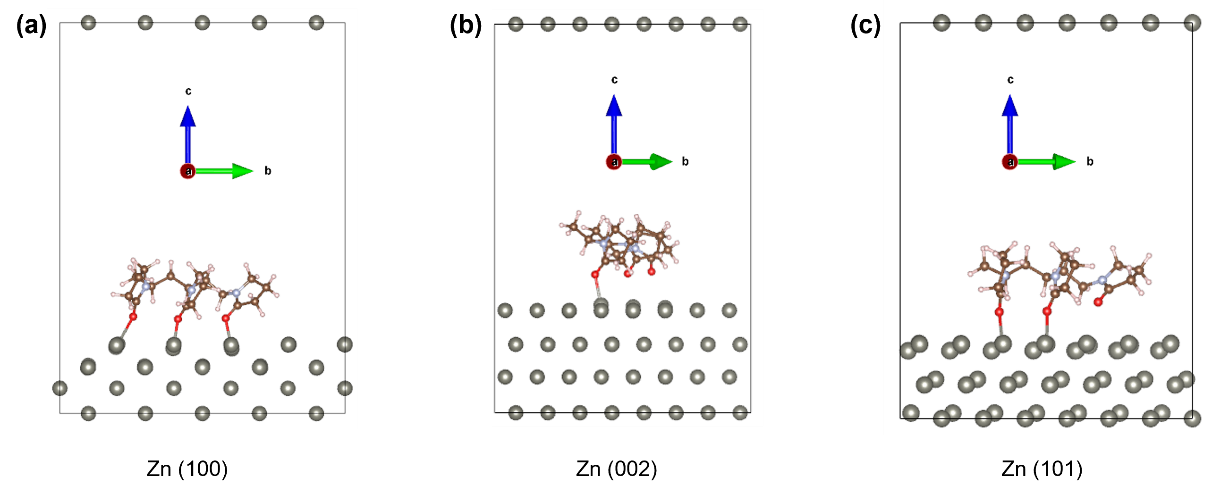


**Figure S3.** Theoretical models of selected capping agent adsorption on (a) Zn (100), (b) Zn (002), and (c) Zn (101) crystal planes.


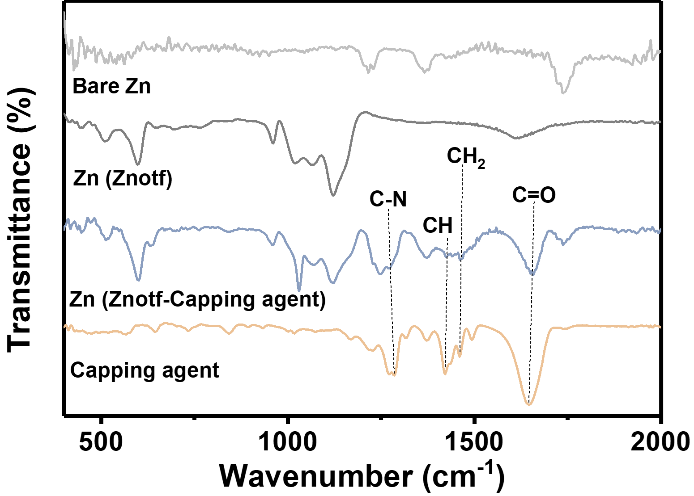


**Figure S4.** FTIR spectra of capping agent solution, bare Zn foil, and washed Zn foils after soaked in different electrolytes.


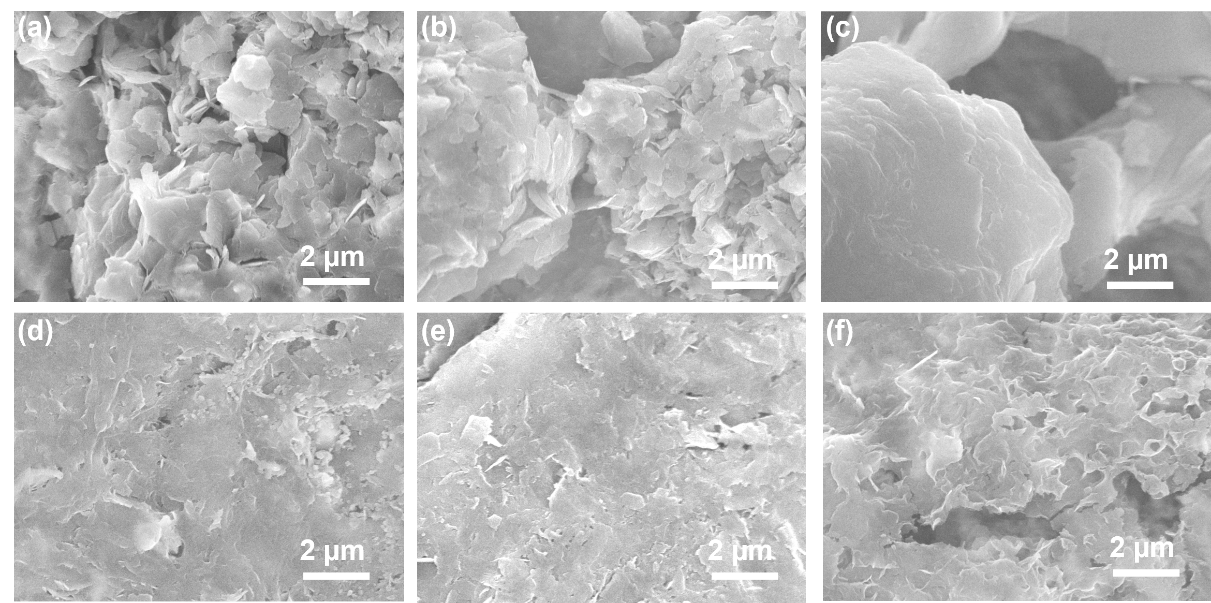


**Figure S5.** (a-c) SEM images of Zn deposition in Znotf electrolyte at a constant areal current density of 1 mA cm^-2^ with different areal capacities of 1 mAh cm^-2^, 5 mAh cm^-2^, and 10 mAh cm^-2^, respectively. (d-f) SEM images of Zn deposition in Znotf-Capping agent electrolyte at a constant areal current density of 1 mA cm^-2^ with different areal capacities of 1 mAh cm^-2^, 5 mAh cm^-2^, and 10 mAh cm^-2^, respectively.


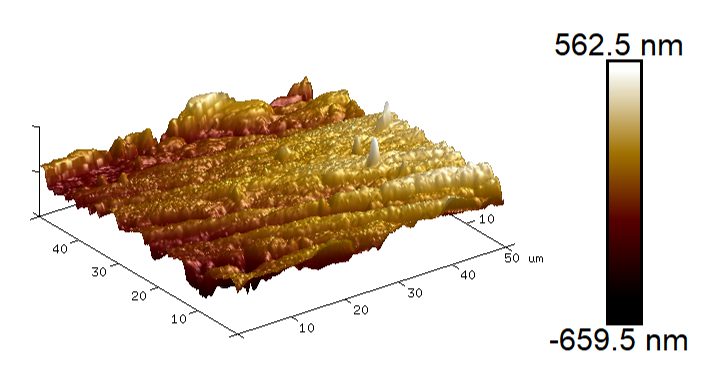


**Figure S6.** AFM image of the bare Zn surface.


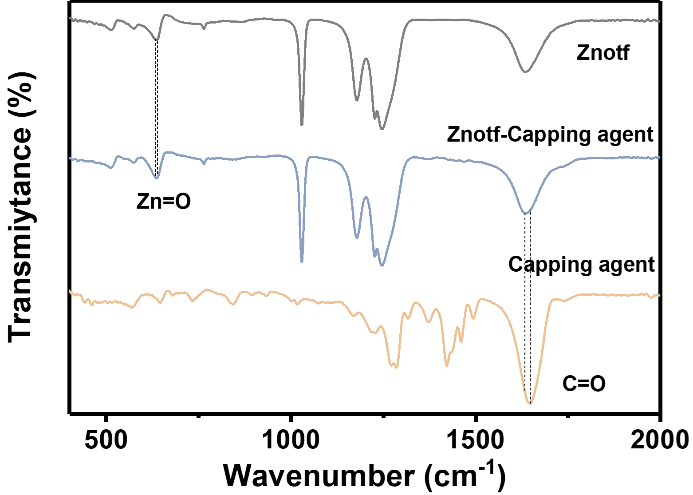


**Figure S7.** FTIR spectra of capping agent solution, Znotf electrolyte, and Znotf-capping agent electrolyte.


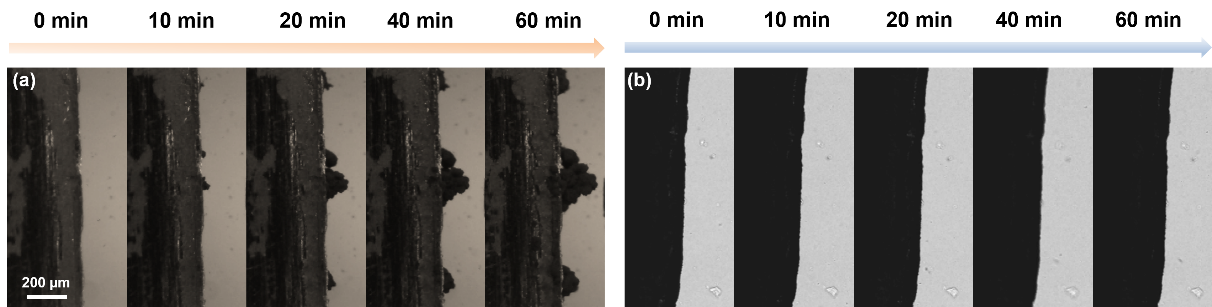


**Figure S8.** (a) In-situ observation of Zn plating in a Zn//Zn cell configuration in Znotf electrolyte. (b) In-situ observation of Zn plating in a Zn//Zn cell configuration in Znotf-Capping agent electrolyte.


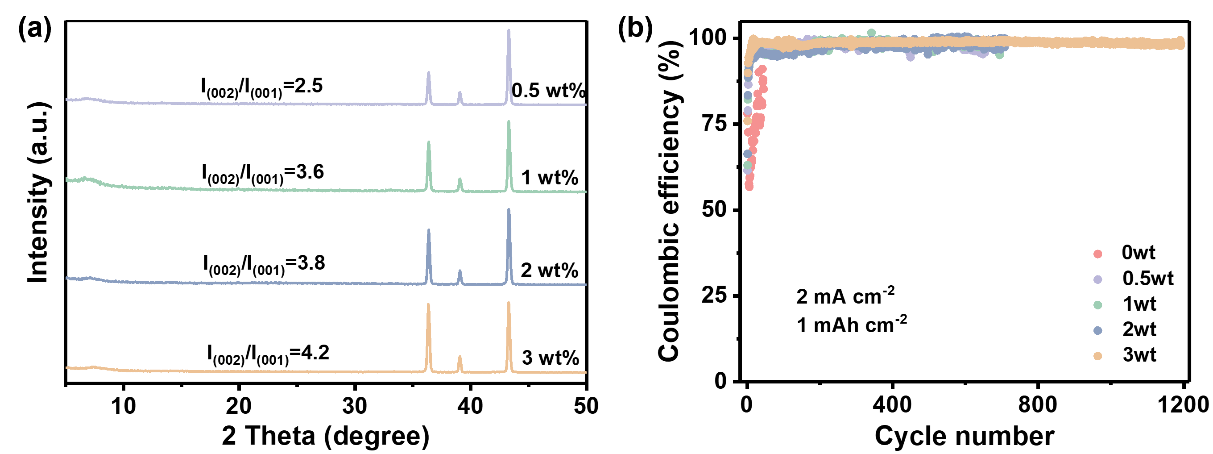


**Figure S9.** (a) XRD patterns of Zn deposition in Znotf electrolyte with different concentrations of the capping agent. (b) Coulombic efficiency of Zn||Cu half cells in Znotf electrolyte and Znotf electrolyte with different concentrations of the capping agent.


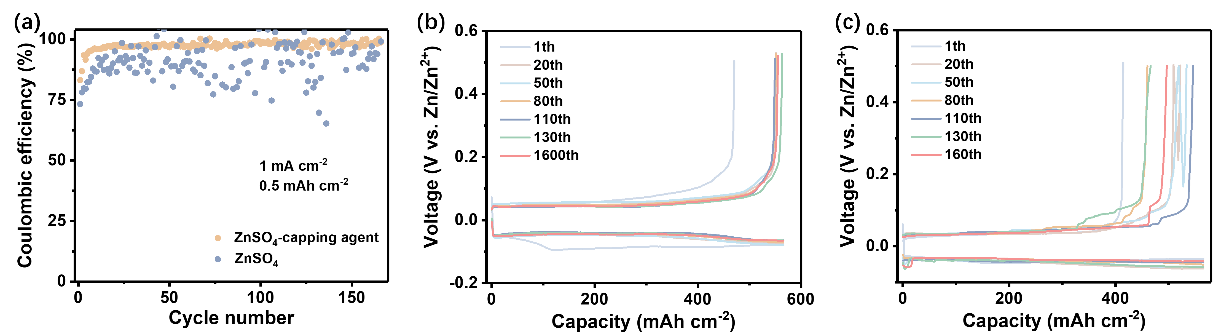


**Figure S10.** Zn plating/stripping behaviors in the ZnSO_4_ and ZnSO_4_-capping agent electrolytes. (a) Coulombic efficiency. (b) Corresponding voltage profiles in the ZnSO_4_-capping agent electrolyte. (c) Corresponding voltage profiles in the ZnSO_4_ electrolyte.


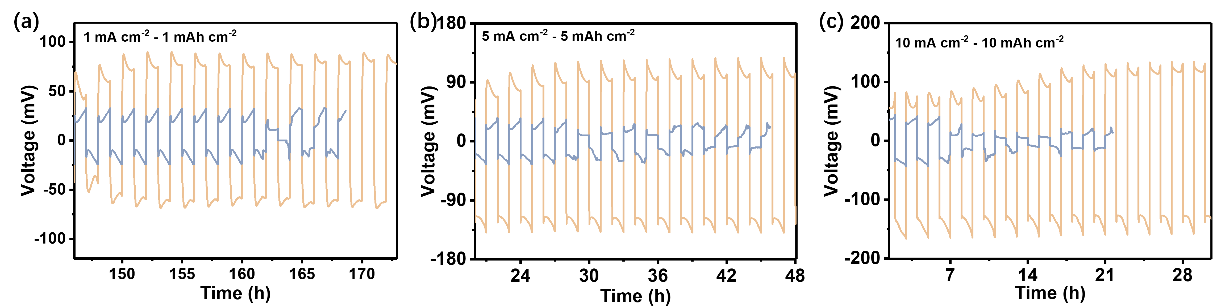


**Figure S11.** Magnified voltage profiles of Zn||Zn symmetric cells at various current densities.


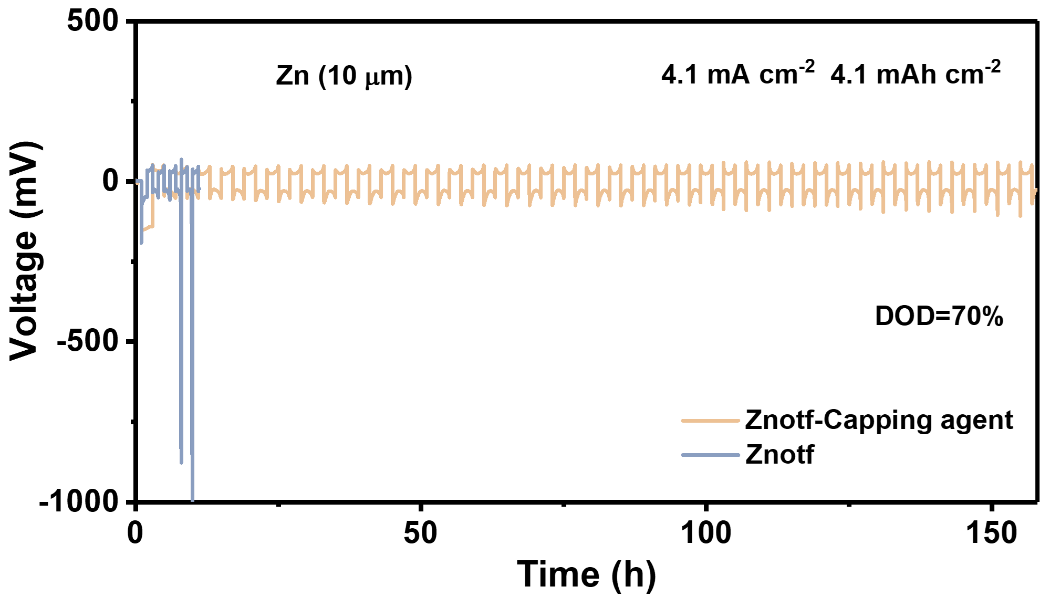


**Figure S12.** Cyclic performance of Zn||Zn symmetric cells at a high Zn utilization rate of 70% depth of discharge (DOD).


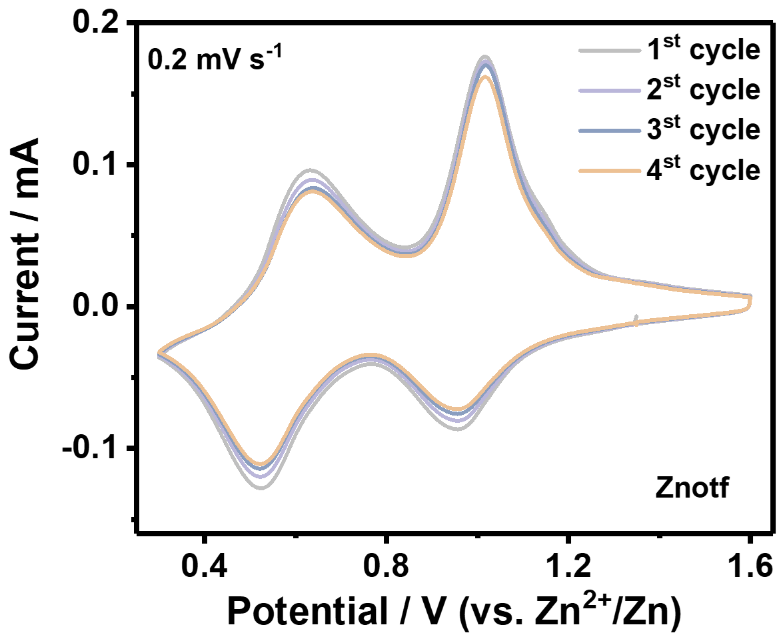


**Figure S13.** CV curves of Zn||V_2_O_5_.xH_2_O batteries at 0.2 mV s^-1^ in Znotf electrolyte.


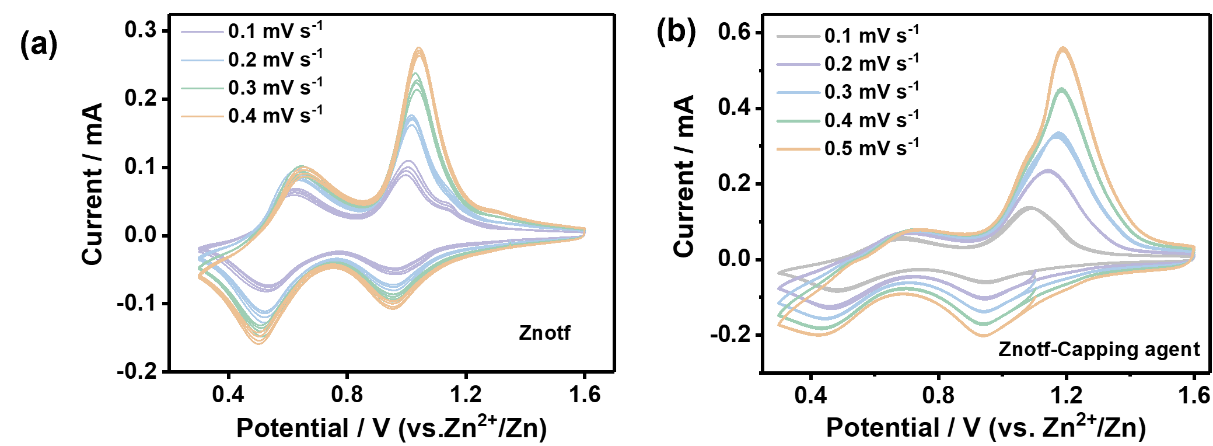


**Figure S14.** (a) CV curves of Zn||V_2_O_5_.xH_2_O batteries at various scan rates in Znotf electrolyte. (b) CV curves of Zn||V_2_O_5_.xH_2_O batteries at various scan rates in Znotf-Capping agent electrolyte.


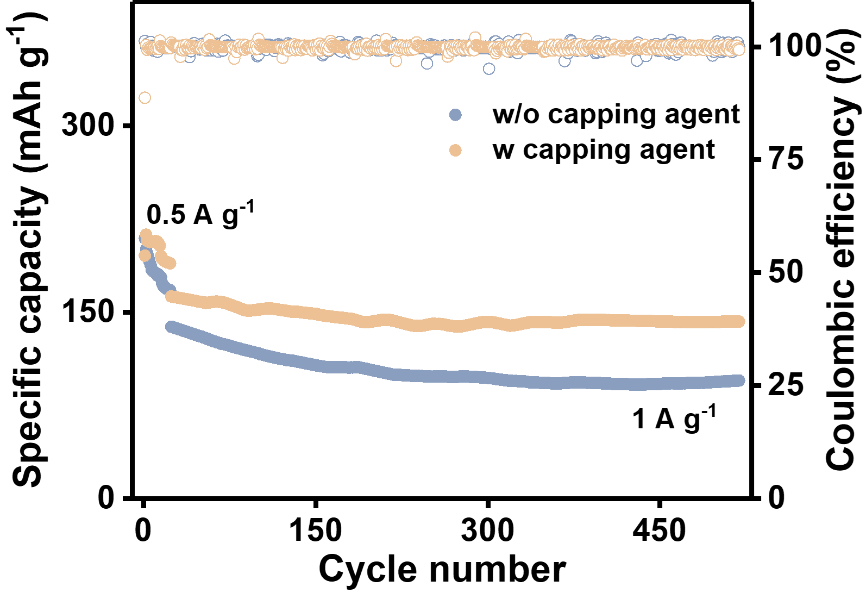


**Figure S15.** Cyclic performance of Zn||MnO_2_ batteries in different electrolytes.


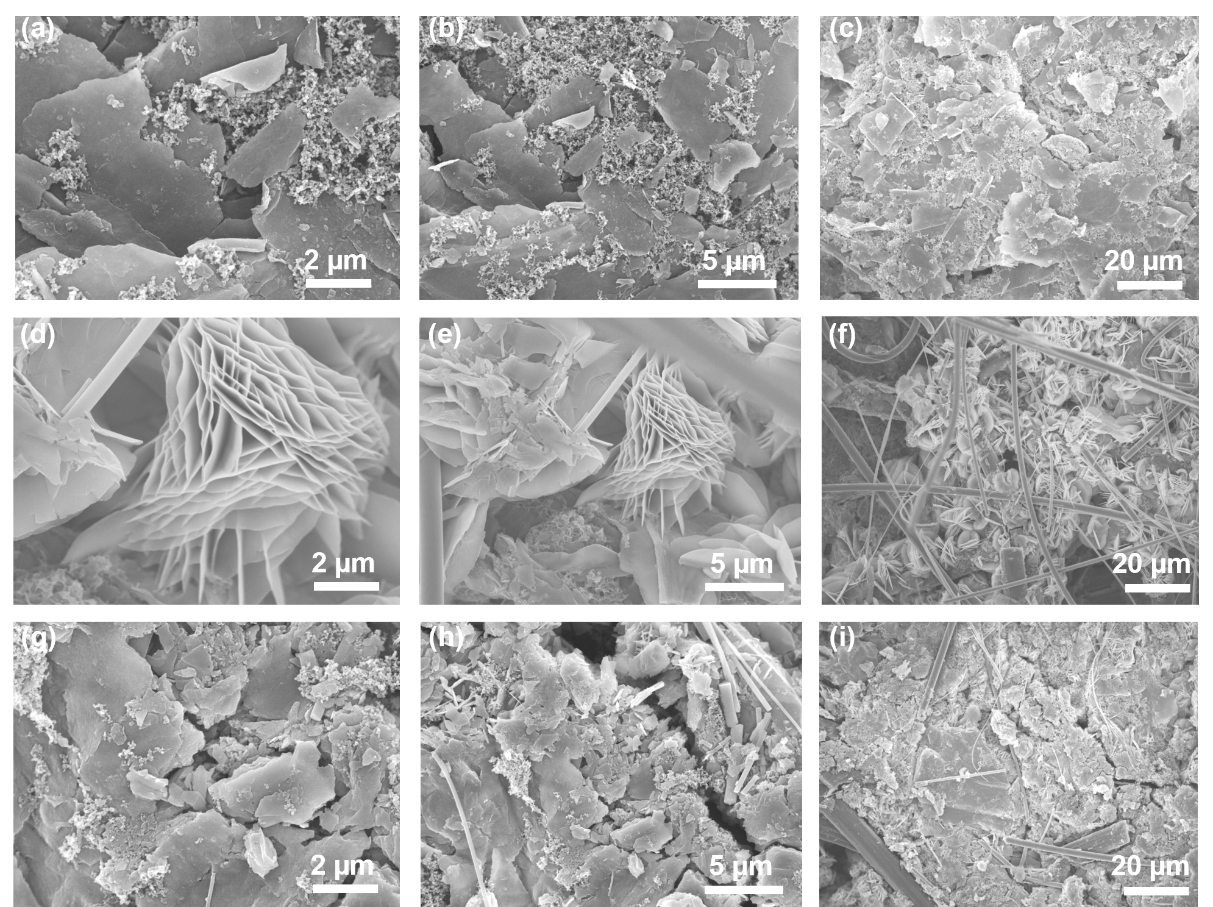


**Figure S16.** (a-c) SEM images of pristine V_2_O_5_.xH_2_O cathode at different magnifications. (d-f) SEM images of V_2_O_5_.xH_2_O cathode after long-term cycling in Znotf electrolyte. (g-i) SEM images of V_2_O_5_.xH_2_O cathode after long-term cycling in Znotf-Capping agent electrolyte.


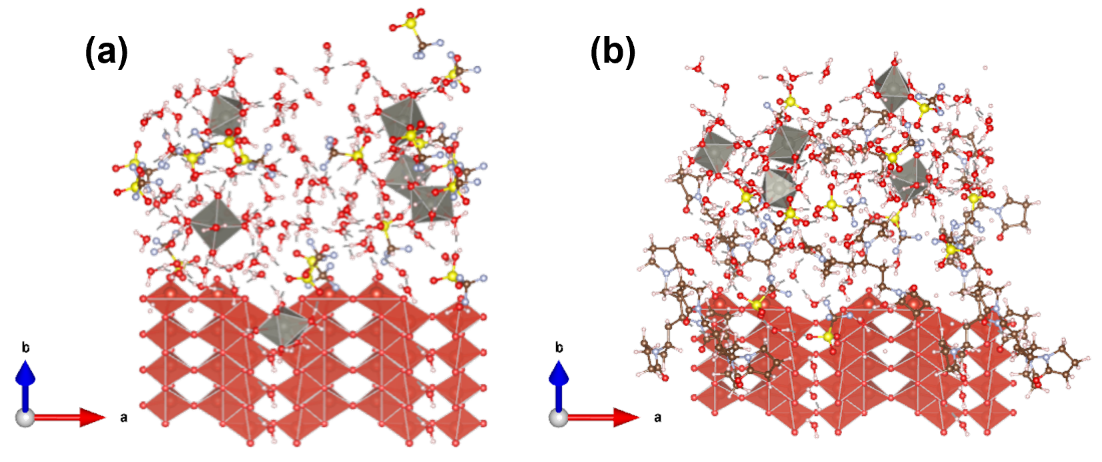


**Figure S17.** (a) Snapshots of electrolyte-cathode interface at the initial stage in Znotf electrolyte. (b) Snapshots of electrolyte-cathode interface at the initial stage in Znotf-capping agent electrolyte.

**Table S1.** Comparison of our work with representative previous reported studies.

| **Ref** | **Material** | **Role** | **Focus** | **Function** | **Electrolyte** | **Cathode** |
| --- | --- | --- | --- | --- | --- | --- |
| 22 | CTAB | Single additive | Anode | Adsorption and dendrite inhibition | ZnSO_4_ | MnO_2_ |
| 23 | PVP | Single additive | Anode and cathode | Less corrosion and uniform Zn striping/plating | ZnSO_4_ | MnO_2_ |
| 24 | PVP | Single additive | Anode | Adsorption and dendrite inhibition | ZnSO_4_ | MnO_2_ |
| 25 | Sodium  citrate | Single additive | Anode | Solvation structure and electrostatic shield | ZnSO_4_ | NaV_3_O_8_^.^1.5H_2_O |
| **This work** | PVP, CTAB, citric acid | Capping agents | Anode, electrolyte, and cathode | Preferred Zn (002) deposition orientation, solvation structure, and CEI | Znotf and ZnSO_4_ | V_2_O_5_^.^xH_2_O, NVO, MnO_2_ |

**Table S2.** RTC values of three dominated crystal planes in different electrolytes.

| **Crystal planes** | **Znotf** | **Znotf-CA** | **Znotf-CTAB** | **Znotf-PVP** |
| --- | --- | --- | --- | --- |
| (002) | 29.8 | 36.5 | 43.8 | 53.7 |
| (100) | 28.2 | 18.2 | 17.1 | 17.2 |
| (101) | 42 | 45.3 | 39.1 | 29.1 |

**Table S3.** Comparison of cyclic stability at high depth of discharge with recently reported studies.

| Number | Depth of discharge | Cyclic time | Ref |
| --- | --- | --- | --- |
| 1 | 88.2 | 70 | Nat. Commun. 2024, 15:302 |
| 2 | 72.17 | 110 | Angew. Chem. 2024, 136, e202407439 |
| 3 | 72.6 | 350 | Adv. Mater. 2024, 36, 2408287 |
| 4 | 72.17 | 110 | Angew. Chem. 2024, 136, e202407439 |
| 5 | 42.7 | 240 | ACS Nano 2023, 17, 11946−11956 |
| 6 | 70 | 100 | Angew. Chem. Int. Ed. 2024, e202415221 |
| 7 | 70 | 160 | This work |

**References**

[1] Y. Kim, Y. Park, M. Kim, J. Lee, K. J. Kim, J. W. Choi, *Nat Commun* **2022**, *13*, 2371.

[2] J. Li, N. Luo, F. Wan, S. Zhao, Z. Li, W. Li, J. Guo, P. R. Shearing, D. J. L. Brett, C. J. Carmalt, G. Chai, G. He, I. P. Parkin, *Nanoscale* **2020**, *12*, 20638-20648.

[3] A. P. Thompson, H. M. Aktulga, R. Berger, D. S. Bolintineanu, W. M. Brown, P. S. Crozier, P. J. in 't Veld, A. Kohlmeyer, S. G. Moore, T. D. Nguyen, R. Shan, M. J. Stevens, J. Tranchida, C. Trott, S. J. Plimpton, Comput. Phys. Commun. 2022, 271, 108171.

[4] G. A. Kaminski, R. A. Friesner, J. Tirado-Rives, W. L. Jorgensen, The Journal of Physical Chemistry B 2001, 105, 6474.

[5] P. Mark, L. Nilsson, J. Phys. Chem. A 2001, 105, 9954.Adv. Mater.

[6] M. J. Frisch, G. W. Trucks, H. B. Schlegel, G. E. Scuseria, M. A. Robb, J. R. Cheeseman, G. Scalmani, V. Barone, G. A. Petersson, H. Nakatsuji, X. Li, M. Caricato, A. V. Marenich, J. Bloino, B. G. Janesko, R. Gomperts, B. Mennucci, H. P. Hratchian, J. V. Ortiz, A. F. Izmaylov, J. L. Sonnenberg, Williams, F. Ding, F. Lipparini, F. Egidi, J. Goings, B. Peng, A. Petrone, T. Henderson, D. Ranasinghe, V. G. Zakrzewski, J. Gao, N. Rega, G. Zheng, W. Liang, M. Hada, M. Ehara, K. Toyota, R. Fukuda, J. Hasegawa, M. Ishida, T. Nakajima, Y. Honda, O. Kitao, H. Nakai, T. Vreven, K. Throssell, J. A. Montgomery Jr., J. E. Peralta, F. Ogliaro, M. J. Bearpark, J. J. Heyd, E. N. Brothers, K. N. Kudin, V. N. Staroverov, T. A. Keith, R. Kobayashi, J. Normand, K. Raghavachari, A. P. Rendell, J. C. Burant, S. S. Iyengar, J. Tomasi, M. Cossi, J. M. Millam, M. Klene, C. Adamo, R. Cammi, J. W. Ochterski, R. L. Martin, K. Morokuma, O. Farkas, J. B. Foresman, D. J. Fox, Gaussian, Inc., Wallingford CT 2016.

[7] a) A. V. Marenich, C. J. Cramer, D. G. Truhlar, J. Phys. Chem. B 2009, 113, 6378; b) J. Zhu, P. Ji, Plasma Phys. Contr. F. 2012, 54, 065004.

[8] a) G. Kresse, J. Hafner, Phys. Rev. B 1994, 49, 14251; b) G. Kresse, J. Furthmüller, Comput. Mater. Sci. 1996, 6, 15.

[9] a) P. E. Blöchl, O. Jepsen, O. K. Andersen, Phys. Rev. B 1994, 49, 16223; b) G. Kresse, D. Joubert, Phys. Rev. B 1999, 59, 1758; c) J. P. Perdew, K. Burke, M. Ernzerhof, Phys. Rev. Lett. 1997, 78, 1396

[10] E. Caldeweyher, C. Bannwarth, S. Grimme, J. Chem. Phys. 2017, 147, 034112.

[11] R. Zhao, X. Dong, P. Liang, H. Li, T. Zhang, W. Zhou, B. Wang, Z. Yang, X. Wang, L. Wang, Z. Sun, F. Bu, Z. Zhao, W. Li, D. Zhao, D. Chao, Adv. Mater. 2023, 35, 2209288.

[12] L. R. De Jesus, G. A. Horrocks, Y. Liang, A. Parija, C. Jaye, L. Wangoh, J. Wang, D. A. Fischer, L. F. Piper, D. Prendergast, Nature communications 2016, 7, 12022.
